# Metabolic and Signaling Dysregulation in a Cellular Model of Hepatic Insulin Resistance

**DOI:** 10.3390/cimb48070729

**Published:** 2026-07-17

**Authors:** Hawraa Zbeeb, Chourouk Joumaa, Giulia De Negri Atanasio, Alberto Diaspro, Laura Vergani

**Affiliations:** 1 Department of Earth, Environment and Life Sciences (DISTAV), University of Genova, Corso Europa 26, 16132 Genova, Italy; hawraa.zbeeb@iit.it (H.Z.); joumaa.chourouk@edu.unige.it (C.J.); giulia.denegri@edu.unige.it (G.D.N.A.); 2 Nanoscopy and NIC@IIT, Istituto Italiano di Tecnologia (IIT), via Enrico melen 83, 16153 Genova, Italy; alberto.diaspro@iit.it; 3 Department of Physics (DIFI), University of Genova, via Dodecaneso 33, 16146 Genova, Italy

**Keywords:** insulin resistance, HepG_2_ cells, hyperglycemia, hyperinsulinemia, free fatty acids, oxidative stress

## Abstract

Insulin resistance (IR) is the underlying pathogenic mechanism for Type 2 Diabetes Mellitus, which is interconnected with Fatty Liver Disease. To investigate the molecular mechanisms by which different metabolic triggers contribute to hepatic IR onset, we exposed human HepG_2_ hepatocytes to varying glucose concentrations (25–50 mM), and/or insulin (1 nM), and a free fatty acid mixture (0.3 mM), mimicking moderate or severe hyperglycemia, hyperinsulinemia, and steatosis. Glucose consumption, glycogen and lipid droplet (LD) accumulation, gene expression, oxidative stress, and insulin signaling were assessed. Under severe hyperglycemia, both insulin and fatty acids decreased glucose consumption, whereas under moderate hyperglycemia, only insulin had this effect. Glycogen accumulation was increased across all treated conditions. Both insulin and fatty acids triggered steatosis and downregulated PPARγ and SIRT1 mRNA, with insulin increasing LD number, while FFAs increased both LD number and size. All conditions enhanced ROS production, resulting in oxidative stress, but with differences in antioxidant enzyme response. Insulin receptor expression was reduced by insulin and FFAs under moderate hyperglycemia but increased by these stimuli under severe hyperglycemia. Both stimuli enhanced AKT phosphorylation under both hyperglycemic conditions. We conclude that different triggers cooperate in promoting hepatic IR through distinct molecular and signaling mechanisms, suggesting that effective treatments may need to target multiple pathways simultaneously.

## 1. Introduction

Insulin resistance (IR) defines a complex metabolic disorder in which insulin fails to elicit the appropriate physiological response in the target tissues. IR is a multifactorial metabolic disorder associated with the development of many diseases, including Type 2 Diabetes Mellitus (T2DM), cardiovascular diseases (CVDs), and certain types of cancer [1]. The liver is the primary source of endogenous glucose production and represents a key insulin target [2]. Indeed, the pathogenesis of IR involves not only increased hepatic glucose production/release but also high levels of circulating free fatty acids (FFAs) and triglycerides (TGs) that contribute to reducing insulin sensitivity, leading to ectopic fat accumulation in the liver and muscle, which further impairs insulin action [3]. For this reason, IR is deeply intertwined in a vicious cycle with metabolic dysfunction-associated steatotic liver disease (MASLD). IR promotes hepatic steatosis through increased hepatic glucose and lipid production, while lipotoxicity associated with MASLD further exacerbates IR. This reciprocal interaction creates a self-perpetuating metabolic dysfunction that often progresses to T2DM [4].

In the liver, insulin binding to the insulin receptor (InsR) triggers the activation of different insulin substrates (IRSs) and the downstream IRS /PI3K/Akt pathway [5]. Insulin signaling leads to suppression of hepatic glucose production by inhibiting the expression of genes responsible for gluconeogenesis. Glycogen synthase kinase-3 (GSK-3) is a constitutively active serine/threonine kinase that exists in two isoforms, GSK-3α and GSK-3β. It is active in resting cells and is inhibited in response to insulin signaling. Dysregulation of GSK-3 activity has been implicated in the development of IR [6]. The phosphoenolpyruvate carboxy kinase 1 (PCK1) is a cytosolic enzyme catalyzing a rate-limiting step in gluconeogenesis to maintain systemic glucose homeostasis. Indeed, PCK1 overactivity contributes to insulin resistance, T2DM and metabolic syndrome [7]. In hepatocytes, the peroxisome proliferator-activated receptor γ (PPARγ) is also involved in regulating insulin sensitivity by controlling the expression of numerous genes that govern glucose and lipid metabolism, and its expression has been directly associated with fatty liver [8]. Another key regulator of glucose and lipid homeostasis is the silent information regulator sirtuin 1 (SIRT1), an NAD^+^-dependent deacetylase, which is downregulated in patients with insulin resistance and T2DM [9].

Oxidative stress, resulting from an imbalance between reactive oxygen species (ROS) production and the antioxidant defense system, plays a central role in the pathophysiology of many diseases, including IR [10,11]. Under physiological conditions, excess ROS are removed by antioxidant enzymes such as cytosolic superoxide dismutase (SOD), which catalyzes O_2_•− conversion to H_2_O_2_, and catalase (CAT), which converts H_2_O_2_ to H_2_O and O_2_. On the other hand, ROS are key signaling molecules playing a crucial role in the progression of inflammation, a common pathogenetic mechanism of many chronic diseases, including diabetes, CVD and cancer [12].

In the literature, most of the in vitro studies for developing IR cellular models have traditionally focused on individual metabolic triggers, such as hyperinsulinemia, hyperglycemia, or hyperlipemia, treating them as isolated insults. Previous reports indicate that elevated levels of insulin (0.1–10 nM) [13,14], glucose (≥55 mM) [15,16], or FFAs (0.25–0.75 mM) [17,18] promote the development and progression of insulin resistance in vitro. However, the molecular mechanisms underlying their combined effects remain poorly understood. Here, we established and characterized distinct cellular models of insulin resistance using human HepG_2_ cells exposed to hyperinsulinemia, hyperglycemia, and hyperlipemia, either individually or in combination. Our comparative approach investigates how distinct IR phenotypes can be developed by HepG_2_ hepatocytes exposed to single or combined metabolic triggers, capturing the distinct adaptive states that emerge following 24 h of trigger exposure without any following stimulation. This approach could allow us to investigate the metabolic reprogramming induced by prolonged exposure to metabolic stressors, and it might serve as a general screening platform, providing a foundation for future detailed molecular and therapeutic studies.

## 2. Materials and Methods

### 2.1. Chemicals

Unless otherwise indicated, the reagents employed were supplied by Sigma-Aldrich Corp. (Milan, Italy).

### 2.2. Cell Culture and Treatment

The human hepatoma cell line HepG_2_ (ATCC HB-8065) was maintained at 37 °C in Dulbecco’s Modified Eagle Medium (DMEM) supplemented with 10% fetal bovine serum (FBS) and 2 mM L-glutamine in a humidified 5% CO_2_ atmosphere. The HepG_2_ line is a well-characterized model for hepatic metabolism studies, retaining key hepatocyte markers while offering high proliferative capacity [19]. For the experiments, once the cells reached 70–80% confluence, the medium was replaced with starvation medium (0.4% FBS and 0.25% BSA). Sterile stock solutions of D-glucose (0.1 g/mL) and insulin (0.5 mM) were prepared and added to the medium to achieve the final concentrations. Stock solutions of sodium oleate (200 mM) and sodium palmitate (43 mM) were prepared and mixed at a 2:1 molar ratio to obtain the steatogenic mixture (0.3 mM). After overnight starvation, the cells were grown for 24 h in the following conditions:

- BG: 5.5 mM glucose—representing physiological glycemia;
- MG: 25 mM glucose—representing moderate hyperglycemia;
- MG + Ins: 25 mM glucose + 1 nM insulin—modeling hyperinsulinemia under moderate hyperglycemia;
- MG + OP: 25 mM glucose + 0.3 mM oleate/palmitate—modeling hepatic steatosis under moderate hyperglycemia;
- HG: 50 mM glucose—representing severe hyperglycemia;
- HG + Ins: 50 mM glucose + 1 nM insulin—modeling hyperinsulinemia under severe hyperglycemia;
- HG + OP: 50 mM glucose + 0.3 mM oleate/palmitate—modeling hepatic steatosis under severe hyperglycemia.

**Figure 1:**
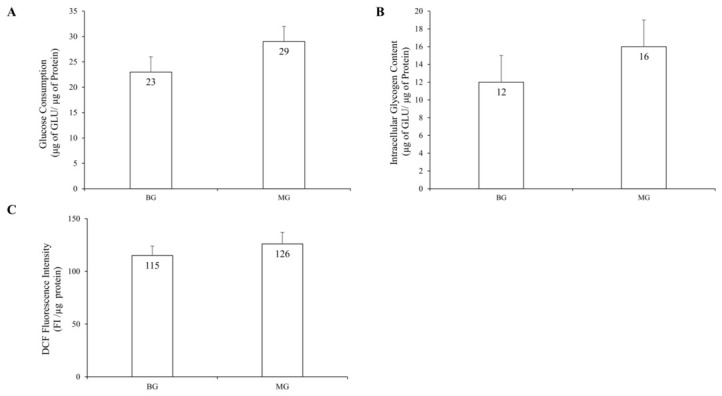
Effects of increasing glucose concentrations in HepG_2_ cells. HepG_2_ cells were treated with a basal physiological glucose concentration (BG, 5.5 mM) or a moderately high glucose concentration (MG, 25 mM) for 24 h. (**A**) Glucose consumption was assessed using the glucose oxidase method. Results are expressed as µg glucose/µg protein after normalization to protein content. (**B**) Intracellular glycogen content was quantified by the phenol–sulfuric acid assay, and results are expressed as µg glucose/µg protein after normalization to protein content. (**C**) Intracellular ROS content was quantified by DCF staining and fluorometric acquisition (λex = 495 nm; λem = 525 nm) using an LS50B fluorimeter (Perkin Elmer, Waltham, MA, USA). Results are expressed as DCF fluorescence intensity (DFI)/µg protein after normalization to protein content. Values represent the mean ± S.D. from at least three independent experiments.

Of note, in preliminary experiments (Figure 1), the physiological glucose concentration (BG) was compared with the moderate glucose concentration (MG), which approximates conditions that stimulate insulin secretion in vivo. No significant differences were observed between these conditions, so MG was selected as the internal control for all subsequent analyses, as it represents the basal condition that promotes insulin release, and BG showed low proliferation compared to the others. All experimental conditions were processed in parallel during each run to ensure consistency and minimize technical variability.

### 2.3. Cell Viability Assay

The possible cytotoxicity of the treatments was tested by the MTT assay based on the reduction of 3-(4,5-dimethylthiazol-2-yl)-2,5-diphenyltetrazolium bromide (MTT) into formazan crystals [20]. Briefly, 1 × 10^4^ cells/well (200 μL) were seeded in a 96-well plate and grown at 37 °C for 24 h. Then, the medium was replaced to start the treatments. At the end of treatments, 20 µL/well of MTT solution (5 mg/mL) was added and incubated for 3 h. Then, the medium was replaced with 200 μL of isopropanol to dissolve the formazan crystals, and the absorbance at 570 nm was measured using a Varian Cary 50 BIO UV-VIS spectrophotometer (Agilent, Milan, Italy).

### 2.4. Glucose Consumption

Glucose consumption by HepG_2_ cells was assessed using the colorimetric glucose oxidase method [21] to quantify the extracellular glucose in the medium. For the experiments, phenol red-free media were used to avoid any interference in spectrophotometric measurements. Briefly, 3 × 10^5^ cells/well were seeded in 6-well plates, with some wells left cell-free to serve as controls. At the end of the treatments, 25 µL of medium was collected from each well and diluted to 3 mL with 0.1 M phosphate buffer (pH 7.0). Then, 1 mL was collected and mixed with 500 µL of enzymatic reagent (prepared by dissolving 1500 U of glucose oxidase, 100 U of glucose peroxidase, 18 mg of 4-aminoantipyrine, and 36 mg of phenol in 100 mL of 0.1 phosphate buffer pH 7.0). Each measurement was duplicated. Samples were incubated at room temperature for 15 min in the dark, and absorbance was then measured at 520 nm using a Varian Cary 50 BIO UV-VIS spectrophotometer (Agilent, Milan, Italy) against a blank (1 mL phosphate buffer and 500 µL enzymatic reagent). A glucose calibration curve (0–30 µg/mL) was preliminarily prepared. Glucose consumption was calculated according to the following equation:

$$\;\left[Glu\;consumbed\right]=\left[Glu\;initial\right]-[\;Glu\;24\;h]$$

where [*Glu initial*] is the glucose concentration in free-cell wells and [*Glu* 24 h] the glucose concentration in the sample after 24 h treatment. The results were normalized to protein content, determined by the Bradford assay, and expressed as µg glucose per µg of protein.

### 2.5. Protein Quantification

The protein content of the samples was quantified by the Bradford assay using BSA as a standard [22].

### 2.6. Intracellular Glycogen Storage

The glycogen content was measured using the Anthrone–Sulfuric Acid method, following the protocol from Teng et al. [23] with slight modifications. At the end of the treatments, HepG_2_ cells were collected, the resulting pellet was resuspended in 500 µL PBS, and an aliquot was taken for protein quantification. Then, the cells were lysed by incubation in 400 µL of 30% KOH for 90 min at 100 °C. After cooling for 10 min, glycogen was precipitated by adding 1.2 mL of absolute ethanol and incubating overnight at −20 °C. After centrifugation at 13,000 rpm for 30 min, the pellet was dried, resuspended in 100 µL of 1 M HCl, and heated at 95 °C for an additional 30 min. Once cooled, 100 µL of 1 M NaOH and 50 µL of dH_2_O were added to each sample. After centrifugation at 13,000 rpm for 10 min, the supernatant was collected and diluted with an equal volume of 5% phenol solution. A glucose calibration curve (0–500 µg/mL) was prepared by mixing 150 µL of each standard with 150 µL of 5% phenol solution. After incubation for 15 min at room temperature, 60 µL of each sample and standard were transferred in triplicate to a 96-well plate, and 150 µL of 96% H_2_SO_4_ was added to each well. A blank was prepared using 60 µL of 5% phenol diluted in water at equal volumes and 150 µL of 96% H_2_SO_4_. After 5 min of incubation, the absorbance at λ = 490 nm was recorded with a Varian Cary 50 BIO UV-VIS spectrophotometer (Agilent, Milan, Italy) against the blank. The results were normalized to protein content and expressed as µg glucose per µg protein.

### 2.7. Intracellular Lipid Accumulation

Neutral lipid accumulation was assessed by Oil Red O (ORO) staining, following a standard protocol [24]. HepG_2_ cells were seeded to achieve approximately 60% confluence at the time of fixation to facilitate image analysis. Upon completion of the experimental treatments, the cells were fixed with 4% paraformaldehyde, rinsed with phosphate-buffered saline (PBS), and stained for 20 min with a 0.3% ORO working solution. This solution was prepared immediately before use by diluting a 0.5% ORO stock solution (in 100% isopropanol) with distilled water at a 3:2 ratio. After staining, the cells were rinsed thoroughly with PBS. Images were acquired using a Leica DMRB light microscope (Leica Microsystems, Milan, Italy) equipped with a Leica DFC420C CCD camera. Image analysis for lipid droplet quantification was performed using ImageJ software (version 2.9.0/1.54c). Lipid droplet area was calculated as the average of all lipid droplets measured from more than four independent sets per condition, and lipid droplet number was normalized to cell number to ensure accurate quantification.

### 2.8. ROS Production

The intracellular level of ROS was quantified in situ using the oxidation of the cell-permeant probe 2′,7′-dichlorofluorescin diacetate (DCF-DA, Fluka, Germany) to the fluorescent compound 2′,7′-dichlorofluorescein (DCF) [25]. Briefly, at the end of the treatments, HepG_2_ cells were collected, centrifuged, and incubated with 1 μM DCF-DA in PBS prepared from a stock solution (10 mM in DMSO) for 30 min at 37 °C in the dark. After that, the cells were centrifuged and suspended in 2 mL PBS. The fluorescence of each sample was read at 25 °C (λex = 495 nm; λem = 525 nm) using a water-thermostatic cuvette holder with an LS50B fluorimeter (Perkin Elmer, Waltham, MA, USA). Results were normalized to protein content and expressed as DCF fluorescence intensity (DFI)/µg protein. The results represent the average of at least three independent biological experiments in triplicate.

### 2.9. RNA Extraction and Real-Time qPCR

Total RNA was extracted from HepG_2_ cells using the TRIZOL reagent (Biorad, Milan, Italy) according to the manufacturer’s instructions. RNA purity was determined by assaying 1 µL of total RNA extract with a UV-VIS spectrophotometer. Total RNA (2 μg) was reverse transcribed using M-MuLV Reverse Transcriptase (Fermentas, Dasit, Italy). The resulting cDNA was used as the template for quantitative real-time PCR (qPCR) to measure mRNA expression levels, as described previously. The qPCR was carried out in quadruplicate for each sample to ensure accuracy and reproducibility, using 1× IQ™ SYBR Green SuperMix and the Chromo4TM System apparatus (Bio-Rad, Milan, Italy), as previously described. Data were calculated by the ΔΔCt method, using glyceraldehyde 3-phosphate dehydrogenase (GAPDH) as the internal control, and then normalized to the control cell value, which was set to be 1. Primer pairs were custom designed specifically for human genes based on their coding sequences, which were obtained from the NCBI GenBank database (http://www.ncbi.nlm.nih.gov/Genbank/, accessed on 1 June 2024). These primers are listed in Table 1.

### 2.10. Western Blot

Western blot was performed as previously described [26] with some modifications. HepG_2_ cells were grown in 100 mm culture dishes until 80% confluency and treated. At the end of the treatments, the cells were collected and lysed with RIPA buffer (50 mM Tris-HCl pH 7.8, 150 mM NaCl, 0.1% Triton X-100, 2.5% SDS, 5 mM DTT, 1 mM EDTA) added with 1 mM PMSF and 1× Protease Inhibitor Cocktail (#P2714-1BTL, Sigma, Milan, Italy). After centrifugation (12,000× *g* for 20 min), aliquots of 15 μg were collected, diluted with RIPA buffer, denatured with SDS sample buffer (95 °C for 10 min), and loaded onto a 10% SDS–PAGE using a standard protein ladder marker (#1610374, BIO-RAD, Milan, Italy). After electrophoresis, proteins were blotted onto a nitrocellulose membrane and stained with Ponceau. The membrane was cut in correspondence with the 55 kDa band, washed and blocked in 5% milk in PBS with 0.15% Tween 20 (PBS-T) for 1 h. The membranes were then incubated overnight at 4 °C with the primary antibody for insulin receptor (rabbit monoclonal antibody #MA5-13783, Thermo Fisher Scientific, Milan, Italy) at a 1:500 dilution, or β-actin (mouse monoclonal antibody #sc-47778, Santa Cruz Biotechnology, Heidelberg, Germany) at a 1:200 dilution, both dissolved in blocking buffer. After three washings with 0.15% PBST at room temperature, the membranes were incubated for 1 h with the secondary antibodies anti-mouse IgG (#A0168 Sigma, Milan, Italy), at a 1:10,000 dilution, or anti-rabbit IgG (#7074B, Cell Signaling, Leiden, The Netherlands), at a 1:2000 dilution, both conjugated to horseradish peroxidase (-HRP). The band corresponding to the full-length InsR was detected at ~110 kDa, consistent with its expected molecular weight. β-actin (45 kDa) served as a loading control to normalize the results. After three washings, chemiluminescence signals were detected using the ChemiDoc™ Touch Imaging System (BioRad, Milan, Italy). Densitometric analysis was performed using ImageJ software (version 2.9.0/1.54c) according to the Hossein Davarinejad protocol [27]. The experiments were performed at least in duplicate on four biological replicates of each set.

### 2.11. Akt Phosphorylation

Akt phosphorylation was measured by ELISA using an Akt (pS473) + total Akt ELISA Kit (Abcam, Cambridge, UK), following the manufacturer’s guidelines. Briefly, an aliquot of 20 μg of each cell lysate was loaded into each well and incubated overnight at 4 °C. Then, the wells were incubated with the primary antibodies against either phospho-AKT (Ser473) or total AKT for 1 h at room temperature. Subsequently, after removing the lysate, 100 μL of HRP-conjugated anti-rabbit IgG was applied for 1 h at room temperature. At the end, 100 μL of TMB One-Step substrate was loaded into each well, and the reaction was allowed to develop for 30 min before the addition of 50 μL of stop solution. The absorbance for each well was recorded immediately at 450 nm, and the data were expressed as the ratio of phosphorylated AKT to total AKT. All measurements were performed at least in duplicate on three biological triplicates of each set.

### 2.12. Statistical Analysis

Data are presented as mean ± S.D. from at least three independent experiments, each conducted in triplicate. All experimental conditions were processed together in the same experimental runs to minimize technical variability and ensure that environmental factors were equally distributed across all groups. Although the experimental design is factorial (glucose × insulin/FFAs), our primary objective was to compare each condition to the MG control to identify significant differences from this reference. Accordingly, the differences among the groups were compared using a one-way analysis of variance (ANOVA) followed by Tukey’s post-test (version 8.0, GraphPad Software, Inc., Boston, MA, USA), which allows comprehensive pairwise comparisons while appropriately controlling the family-wise error rate.

## 3. Results

### 3.1. Setting and Characterization of the HepG_2_ Model of IR

**Figure 2:**
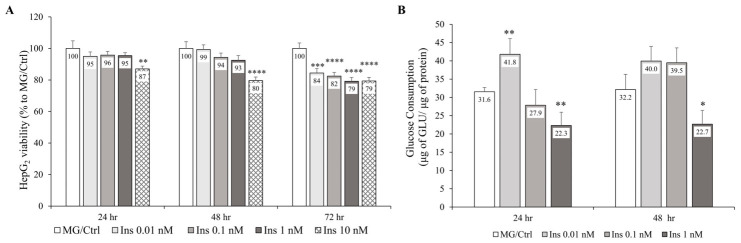
Effects of increasing insulin concentration on glucose consumption in HepG_2_ cells as a model of insulin resistance. (**A**) HepG_2_ cell viability upon exposure to increasing concentrations of insulin (0.01–10 nM) for 24, 48, and 72 h, assessed by the MTT assay. (**B**) Glucose consumption in HepG_2_ cells exposed to increasing insulin concentrations for 24 and 48 h using the glucose oxidase method. Results are expressed as µg of glucose per µg of protein (µg glucose/µg protein). Values represent the mean ± S.D. from at least three independent experiments. Statistical significance between groups was assessed using ANOVA followed by Tukey’s test. Symbols: vs. MG, * *p* ≤ 0.05, ** *p* ≤ 0.01, *** *p* ≤ 0.001, and **** *p* ≤ 0.0001.

To mimic moderate and severe hyperglycemia, HepG_2_ cells were grown under moderate (25 mM glucose, MG) and severe (50 mM glucose, HG) glucose concentrations. We initially performed a titration experiment using increasing insulin concentrations (0–10 nM) over 24, 48 and 72 h in cells maintained in MG, which served as the internal control modeling the in vivo condition in which insulin is secreted in response to rising blood glucose levels. The MTT assay was performed to evaluate the possible cytotoxicity of excess insulin on HepG_2_ cells (Figure 2A). No significant toxicity was detected for the lowest insulin concentrations (0.01–1 nM) at both 24 and 48 h, whereas 10 nM insulin significantly reduced cell viability (approximately −13% and −20% at 24 and 48 h, respectively). At 72 h, all insulin concentrations showed significant toxicity.

As an initial rapid and reliable screening step, we measured glucose consumption from the culture medium, a well-established marker of impaired glucose handling in insulin resistance, in HepG_2_ cells maintained under MG conditions with varying insulin concentrations to determine the optimal dose for subsequent experiments (Figure 2B). After 24 h, 0.01 nM insulin increased glucose consumption (+10 µg glucose/µg protein vs. MG; *p* ≤ 0.05), while 1 nM insulin significantly decreased it (−9.2 µg glucose/µg protein at 24 h and −9.5 µg glucose/µg protein at 48 h vs. MG; *p* ≤ 0.05). Based on these results, we adopted a 24 h treatment with 1 nM insulin to model IR in vitro in HepG_2_ cells.

**Figure 3:**
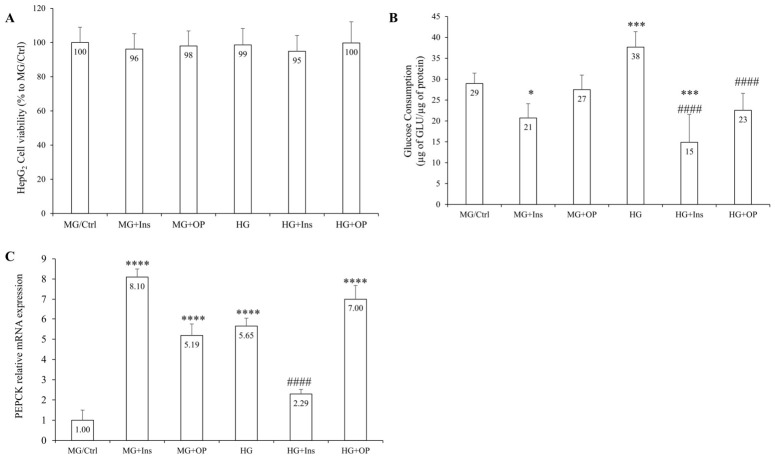
Effects of insulin, FFAs, and glucose on glucose consumption in HepG_2_ cells as a model of insulin resistance. (**A**) HepG_2_ cell viability after exposure to metabolic stressors for 24 h was assessed by the MTT assay. (**B**) Glucose consumption was assessed using the glucose oxidase method in HepG_2_ cells incubated under MG (25 mM internal control) or HG (50 mM) glucose concentrations in the presence or absence of insulin (1 nM) and FFAs (0.3 mM) for 24 h. Results are expressed as µg glucose/µg protein after normalization to protein content. (**C**) PCK1 mRNA expression was quantified by RT-qPCR using GAPDH as an internal control and expressed as fold change relative to the MG sample. Values represent the mean ± S.D. from at least three independent experiments. Statistical significance between groups was assessed using ANOVA followed by Tukey’s test. Symbols: vs. MG, * *p* ≤ 0.05, *** *p* ≤ 0.001, and **** *p* ≤ 0.0001; vs. HG, #### *p* ≤ 0.0001.

Elevated circulating FFAs contribute to insulin resistance in vivo. Therefore, we exposed HepG_2_ cells to 0.3 mM FFAs (OP) under both MG and HG conditions to model the effects of hyperlipemia under moderate and severe hyperglycemic conditions, respectively. The MTT assay excluded any significant toxicity of all treatments after 24 h (Figure 3A). No significant difference in glucose consumption was detected between BG and MG (Figure 1A). Conversely, HG stimulated glucose consumption by +8 µg glucose/µg protein (*p* ≤ 0.001) compared to the internal control MG (Figure 3B). Interestingly, under MG conditions, insulin (MG + Ins) significantly reduced glucose consumption (−7 µg glucose/µg protein vs. MG; *p* ≤ 0.05), whereas excess FFAs (MG + OP) had no effect. In contrast, under HG conditions, glucose consumption was significantly impaired by both insulin (HG + Ins) and FFAs (HG + OP) (−23 and −14 µg glucose/µg protein vs. HG, respectively; *p* ≤ 0.05).

Hepatic PCK1 (PEPCK) controls internal glucose production to maintain systemic glucose homeostasis. PCK1 mRNA expression was quantified by real-time qPCR (Figure 3C). A significant upregulation of PCK1 mRNA was observed in HG compared to MG (5.65-fold; *p* ≤ 0.05). Moreover, PCK1 mRNA was upregulated in MG + Ins (8.10-fold; *p* ≤ 0.05) and in MG + OP cells (5.19-fold; *p* ≤ 0.05) compared with the internal control MG. Of note, under HG conditions, insulin significantly reduced PCK1 expression (0.41-fold vs. HG; *p* ≤ 0.05), whereas FFAs maintained PCK1 expression at levels comparable to HG alone.

Taken together, these findings suggest that hyperinsulinemia induces the insulin resistance phenotype regardless of glucose excess, while excess FFAs contribute to IR primarily under conditions of severe hyperglycemia.

### 3.2. IR In Vitro Promotes Glycogen and Triglyceride Accumulation in HepG_2_ Cells

**Figure 4:**
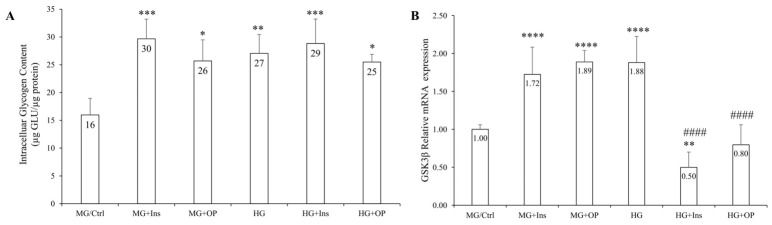
Effects of insulin, FFAs, and glucose on intracellular glycogen storage and GSK-3β expression. HepG_2_ cells were incubated under MG (25 mM internal control) or HG (50 mM) glucose concentrations in the presence or absence of insulin (1 nM) and FFAs (0.3 mM) for 24 h. (**A**) Intracellular glycogen content was quantified by the phenol–sulfuric acid assay, and results are expressed as µg glucose/µg protein after normalization to protein content. (**B**) GSK-3β mRNA expression was quantified by RT-qPCR using GAPDH as an internal control and expressed as a fold change relative to the MG sample. Values represent the mean ± S.D. from at least three independent experiments. Statistical significance between groups was assessed using ANOVA followed by Tukey’s test. Symbols: vs. MG, * *p* ≤ 0.05, ** *p* ≤ 0.01, *** *p* ≤ 0.001, and **** *p* ≤ 0.0001; vs. HG, #### *p* ≤ 0.0001.

To assess the effects of metabolic stressors on downstream utilization of glucose following its uptake, intracellular glycogen accumulation was measured (Figure 4A). Similar to glucose consumption, no significant difference in glycogen content was detected between BG and MG (Figure 1B). Compared to the internal control (MG), all treatments significantly stimulated glycogen accumulation. Under MG conditions, both insulin (MG + Ins) and FFAs (MG + OP) significantly enhanced intracellular glycogen accumulation (+13 and +9 µg glucose/µg protein vs. MG, respectively; *p* ≤ 0.05). Notably, severe hyperglycemia (HG) resulted in a significant increase in glycogen accumulation compared with MG (+15 µg glucose/µg protein; *p* ≤ 0.05). However, under HG conditions, neither insulin (HG + Ins) nor FFAs (HG + OP) increased glycogen accumulation compared to HG alone.

To further investigate the molecular mechanisms underlying the observed changes in glycogen accumulation, GSK-3β mRNA expression was quantified by real-time qPCR (Figure 4B). A significant upregulation of GSK-3β mRNA was observed in HG compared to MG (1.88-fold; *p* ≤ 0.05), as well as in MG cells treated with insulin (MG + Ins) or FFAs (MG + OP) (1.72- and 1.89-fold, respectively; *p* ≤ 0.05). Conversely, although HG alone significantly increased GSK-3β mRNA levels relative to MG, exposure of HG cells to either insulin or FFAs resulted in a significant downregulation of GSK-3β mRNA (0.27- and 0.43-fold relative to HG, respectively; *p* ≤ 0.05), reaching levels comparable to those observed in MG.

**Figure 5:**
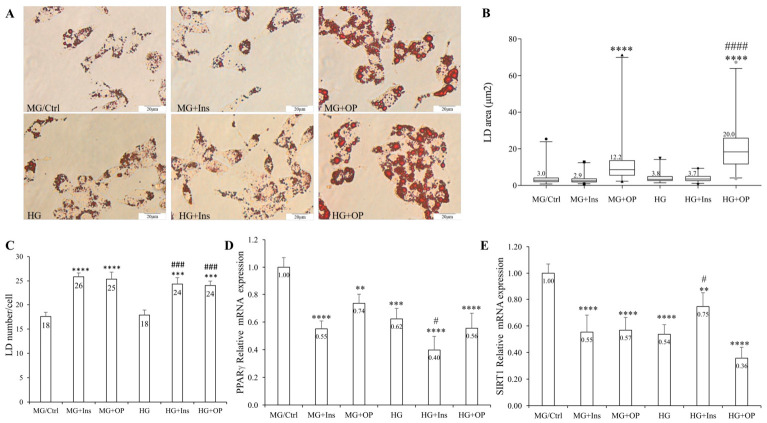
Effects of insulin, FFAs, and glucose on intracellular lipid accumulation. HepG_2_ cells were incubated under MG (25 mM internal control) or HG (50 mM) glucose concentrations in the presence or absence of insulin (1 nM) and FFAs (0.3 mM). (**A**) Lipid droplet (LD) accumulation was visualized by ORO staining and optical microscopy using a Leica DMRB light microscope (40× magnification; scale bar: 20 µm). ImageJ software was used for quantitative image analysis to determine (**B**) the number of LDs per cell (box and whisker plots show the median (center line), 25th–75th percentiles (box), and 1st–99th percentiles (whiskers). Values outside the 1st–99th percentile range are shown as individual outliers) and (**C**) the average size of LDs. Additionally, mRNA expression of (**D**) PPARγ and (**E**) SIRT1 was quantified by RT-qPCR using GAPDH as an internal control and expressed as fold change relative to the MG sample. Values represent the mean ± S.D. from at least three independent experiments. Statistical significance between groups was assessed using ANOVA followed by Tukey’s test. Symbols: vs. MG, ** *p* ≤ 0.01, *** *p* ≤ 0.001, and **** *p* ≤ 0.0001; vs. HG, # *p* ≤ 0.05, ### *p* ≤ 0.001, and #### *p* ≤ 0.0001.

Given the close association between insulin resistance and hepatic steatosis, we examined triglyceride (TG) accumulation in cytosolic lipid droplets (LDs) under the different treatment conditions using ORO staining (Figure 5A–C). When the internal control MG was exposed to FFAs, we observed a significant increase in both the number (+7 LDs/cell) and average size (+9.2 µm^2^) of LDs compared to MG (*p* ≤ 0.05). A similar effect was observed under HG conditions, where FFAs increased LD number (+6 LDs/cell) and size (+16.2 µm^2^) compared to HG (*p* ≤ 0.05). Interestingly, under both MG and HG conditions, insulin increased LD number (+8 and +6 LDs/cell, respectively; *p* ≤ 0.05) without affecting LD size.

In the human liver, PPARγ acts as a master regulator of lipogenesis and TG accumulation. As shown in Figure 5D, PPARγ mRNA was downregulated in HG compared to the internal control MG (0.62-fold; *p* ≤ 0.05), as well as in MG cells exposed to either insulin (0.55-fold; *p* ≤ 0.05) or FFAs (0.74-fold; *p* ≤ 0.05). Also under HG conditions, insulin further downregulated PPARγ expression (0.40-fold vs. HG; *p* ≤ 0.05). SIRT1 is another key regulator of hepatic metabolic homeostasis and is often associated with improved insulin sensitivity and lipid metabolism. As shown in Figure 5E, SIRT1 mRNA was downregulated in HG compared to the internal control MG (0.54-fold; *p* ≤ 0.05), as well as in MG cells exposed to either insulin or FFAs (0.55- and 0.57-fold, respectively; *p* ≤ 0.05). Of note, under HG conditions, insulin partially restored SIRT1 expression (1.39-fold vs. HG; *p* ≤ 0.05), whereas FFAs further exacerbated its downregulation (0.67-fold vs. HG; *p* ≤ 0.05).

### 3.3. IR In Vitro Stimulates ROS Production and Antioxidant Defense in HepG_2_ Cells

**Figure 6:**
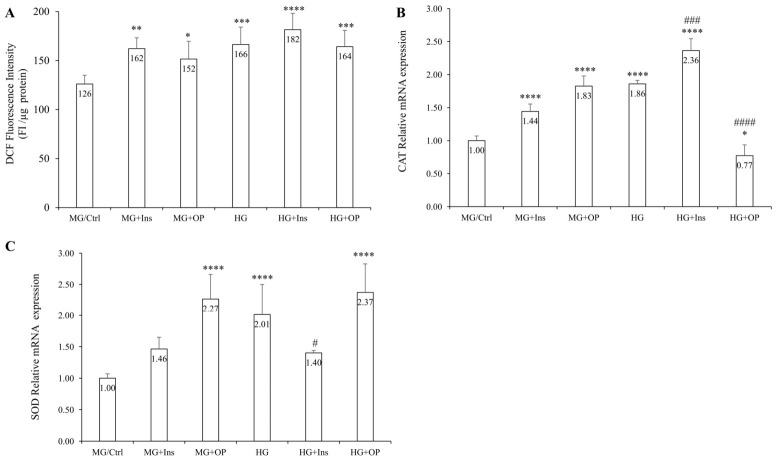
Effects of insulin, FFAs, and glucose on oxidative stress. HepG_2_ cells were incubated under MG (25 mM internal control) or HG (50 mM) glucose concentrations in the presence or absence of insulin (1 nM) and FFAs (0.3 mM). (**A**) Intracellular ROS content was quantified by DCF staining and fluorometric acquisition (λex = 495 nm; λem = 525 nm) using an LS50B fluorimeter. Results are expressed as DCF fluorescence intensity (DFI)/ µg protein after normalization to protein content. mRNA expression of the antioxidant enzymes (**B**) CAT and (**C**) SOD was quantified by RT-qPCR, normalized to GAPDH, and expressed as fold change relative to the internal control MG. Values represent the mean ± S.D. from at least three independent experiments. Statistical significance between groups was assessed using ANOVA followed by Tukey’s test. Symbols: vs. MG, * *p* ≤ 0.05, ** *p* ≤ 0.01, *** *p* ≤ 0.001, and **** *p* ≤ 0.0001; vs. HG, # *p* ≤ 0.05, ### *p* ≤ 0.001 and #### *p* ≤ 0.0001.

Successively, we investigated whether exposure to the various metabolic stressors triggers oxidative stress in HepG_2_ cells. Intracellular ROS production was measured by DCF staining (Figure 6A). All treatments, compared to the internal control MG, induced a significant increase in ROS production. Specifically, HG markedly stimulated ROS production compared to the internal control MG (+52 DFI/µg protein; *p* ≤ 0.05). Under MG conditions, both insulin and FFAs significantly increased ROS production (+36 and +25 DFI/µg protein vs. MG, respectively; *p* ≤ 0.05). In contrast, under HG conditions, neither insulin nor FFAs further altered ROS levels.

To gain further mechanistic insight, we assessed the mRNA expression of the main antioxidant enzymes, catalase (CAT) and superoxide dismutase (SOD), by qPCR (Figure 6B,C). HG markedly upregulated both CAT and SOD mRNA compared to the internal control MG (1.86- and 2.01-fold, respectively; *p* ≤ 0.05). Under MG conditions, insulin selectively upregulated CAT (1.4-fold; *p* ≤ 0.05) without affecting SOD, whereas FFAs upregulated both enzymes (1.8- and 2.27-fold, respectively; *p* ≤ 0.05). Under HG conditions, insulin further upregulated CAT (1.27-fold vs. HG; *p* ≤ 0.05) while suppressing SOD expression (0.7-fold vs. HG; *p* ≤ 0.05), while FFAs suppressed CAT expression (0.42-fold vs. HG; *p* ≤ 0.05) without affecting SOD.

### 3.4. IR In Vitro Impacts the Insulin Signaling Pathway in HepG_2_ Cells

**Figure 7:**
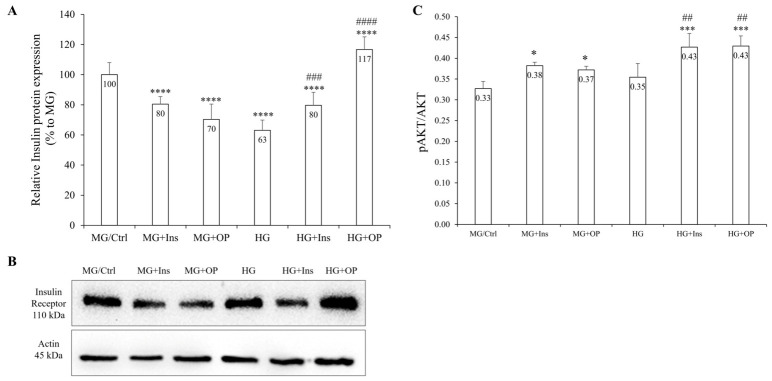
Effects of insulin, FFAs, and glucose on the insulin signaling pathway. HepG_2_ cells were incubated under MG (25 mM internal control) or HG (50 mM) glucose concentrations in the presence or absence of insulin (1 nM) and FFAs (0.3 mM). (**A**) Densitometric analysis of InsR expression normalized to actin. Experiments were performed in duplicate across four biological replicates (n = 4). Results are expressed as the mean InsR/actin ratio quantified using ImageJ software and presented as a percentage relative to MG. (**B**) Representative blots showing InsR (110 kDa) and actin (45 kDa). The membrane was sectioned horizontally at ~55 kDa after blotting onto a nitrocellulose membrane, and the resulting sections were probed independently. Images are original acquisitions from the same membrane. Cropping was performed solely to remove the non-informative background; no bands were excluded. Full-length images are provided in the Supplementary Figure S1. (**C**) The ratio of phosphorylated AKT (Ser473) to total AKT (pAKT/AKT) was quantified using a commercially available ELISA kit. Values represent the mean ± S.D. from at least three independent experiments. Statistical significance between groups was assessed using ANOVA followed by Tukey’s test. Symbols: vs. MG, * *p* ≤ 0.05, *** *p* ≤ 0.001, and **** *p* ≤ 0.0001; vs. HG, ## *p* ≤ 0.01, ### *p* ≤ 0.001, and #### *p* ≤ 0.0001.

Insulin receptor (InsR) expression was quantified by Western blot (Figure 7A,B). Compared to the internal control MG, a marked downregulation of InsR was detected in HG cells (−37% vs. MG; *p* ≤ 0.05). Under MG conditions, exposure to either insulin or FFAs significantly reduced InsR levels (−20% and −30% vs. MG, respectively; *p* ≤ 0.05). Strikingly, this pattern was reversed under HG conditions, where both insulin and FFAs increased InsR level by 26% and 85%, respectively, compared with HG alone (*p* ≤ 0.05).

Finally, we assessed downstream effects of insulin receptors by measuring AKT phosphorylation via ELISA (pAKT/AKT ratio; Figure 7C). Unlike InsR expression, the pAKT/AKT ratio showed no significant difference between MG and HG conditions (0.31 ± 0.01 and 0.35 ± 0.03, respectively). However, both insulin and FFAs markedly stimulated AKT phosphorylation in both glucose environments. Under MG conditions, the pAKT/AKT ratio was significantly increased by both insulin and FFAs compared to MG alone (0.38 ± 0.01 and 0.37 ± 0.01, respectively; *p* ≤ 0.05). This increase was even more pronounced under HG conditions (0.41 ± 0.02 for insulin and 0.43 ± 0.03 for FFAs; *p* ≤ 0.05).

## 4. Discussion

In the present study, we established and characterized an in vitro model of hepatic insulin resistance using human HepG_2_ cells to dissect the distinct contributions of high levels of glucose, insulin, and fats. These findings indicate that, while hyperglycemia, hyperinsulinemia, and hyperlipemia all elicit an IR phenotype in HepG_2_ cells, each operates through a distinct molecular mechanism.

To establish a reliable and accurate in vitro model of insulin resistance, we first characterized the insulin dose–response in HepG_2_ cells under moderate (25 mM) hyperglycemia, where the MG condition served as the internal control in all experiments. Of note, at a low dose (0.01 nM), insulin stimulated glucose consumption in HepG_2_ cells, thus confirming the insulin-responsive phenotype of these hepatoma cells. We wish to emphasize that HepG_2_ cells are known to retain many metabolic features of human hepatocytes, and importantly, their insulin-resistant phenotype can recapitulate key aspects of the insulin-resistant state observed in vivo [28]. Accordingly, the higher insulin concentration (1 nM) blunted the response in HepG_2_ cells, inducing a state that mimics IR in vitro. This insulin concentration was therefore selected to model hepatic IR in our subsequent experiments. Based on the known contributions of metabolic stressors to IR development and establishment [29,30], we also evaluated the effects of glucose and FFA overload in HepG_2_ cells. We observed that IR induction is context-dependent: in fact, insulin promoted IR characteristics under both moderate and severe hyperglycemia, whereas FFA overload exacerbated IR characteristics only in the presence of severe hyperglycemia, highlighting a deleterious interaction between nutrient excesses.

Glycogen content is mechanistically linked to glucose uptake and insulin resistance in hepatic cells. During insulin resistance, glycogen metabolism is dysregulated, with altered glycogen synthase (GS) activity [31]. Of note, we observed increased glycogen accumulation across all conditions. In the context of glycogen metabolism, GSK-3β serves as a key regulator of glycogen synthesis by inactivating glycogen synthase [6]. Interestingly, despite the increased glycogen storage observed under moderate glucose conditions, GSK-3β mRNA was upregulated by insulin and fatty acids, as well as by high glucose. In contrast, under severe hyperglycemia, both insulin and fatty acids downregulated GSK-3β mRNA, suggesting a context-dependent regulation of glycogen metabolism. This apparent discrepancy is resolved by recognizing that GSK-3β activity is predominantly regulated at the post-translational level via AKT-mediated inhibitory phosphorylation at Ser9, downstream of AKT phosphorylation at Ser473 [32,33]. Accordingly, despite increased GSK-3β mRNA, the elevated pAKT (Ser473) levels observed in our study likely resulted in GSK-3β inactivation, overriding transcriptional regulation and thereby contributing to the increased glycogen content. Moreover, the AKT/PPP1R3G axis promotes glycogen synthesis in parallel to GSK-3β, promoting glycogen deposition even when classical insulin signaling is impaired [34]. Also, severe hyperglycemia itself allosterically activates GS, bypassing insulin signaling [35]. Thus, in HepG_2_ cells, elevated glycogen content can occur despite reduced glucose uptake, reflecting the engagement of **these** alternative pathways that uncouple glycogen storage from acute insulin responsiveness.

Dysregulation of gluconeogenesis is a hallmark of hepatic IR, with PCK1 (PEPCK) integrating hormonal and nutritional signals [36]. In our model, glucose consumption and PCK1 expression exhibited an inverse but context-dependent relationship. Under moderate hyperglycemia, insulin reduced glucose consumption while upregulating PCK1, reflecting a classical IR phenotype where hyperinsulinemia fails to suppress gluconeogenic genes [18] and reduced glucose uptake and deficiency in cells trigger compensatory PCK1 upregulation [37]. In contrast, PCK1 upregulation during FFA overload aligns with its role in providing glycerol-3-phosphate for triglyceride synthesis [38] rather than gluconeogenic activation, consistent with the observed lipid accumulation. Under severe hyperglycemia, insulin blunted the compensatory PCK1 response, revealing a distinct insulin-resistant phenotype.

On the other hand, insulin promotes fat deposition in LDs, and IR is a pathophysiological factor in MASLD [39]. High levels of circulating FFAs are associated with IR in both animals and humans [40]. Our observations reveal distinct regulatory roles for glucose, insulin and FFAs in LD dynamics. While FFA excess robustly increased both LD number and size, insulin increased LD number without affecting size. This supports the model that insulin promotes the de novo formation of LDs, rather than their expansion, by driving lipogenesis through the coordinated upregulation of glucokinase (GK) and lipogenic enzymes such as fatty acid synthase (FAS) and acetyl-CoA carboxylase (ACC), which together convert glucose-derived precursors into fatty acids [41,42]. In parallel, we also investigated PPARγ, the main lipogenic transcription factor in human hepatocytes. Previous evidence suggested that PPARγ induction alleviated IR [43], and mice with increased PPARγ activity were protected from obesity-induced IR, whereas mice lacking PPARγ developed hyperlipidemia, hyperglycemia, or hyperinsulinemia [44]. Our findings are consistent with impaired PPARγ function in the hepatic IR model, showing that severe hyperglycemia, as well as insulin and FFAs, downregulated PPARγ mRNA expression in HepG_2_ cells. Although mRNA expression of PPARγ was downregulated as expected, we acknowledge that PPARγ function is primarily regulated at the protein level; however, the observed lipid accumulation phenotype supports a functional role for PPARγ dysregulation in our model.

Hyperglycemia is known to stimulate ROS production, resulting in oxidative stress, which sustains IR and T2DM pathogenesis [45,46]. Our data reveal interesting new insights. Under MG conditions, both insulin and FFAs stimulate ROS production, whereas under HG, this prooxidant effect is lost. Likely, at the cellular level, severe hyperglycemia leads to a state of maximal oxidative stress, resulting in a “ceiling effect” that masks the further contributions of insulin and FFAs. Intracellular ROS are scavenged by various enzymes. Our findings show that glucose, FFAs and insulin differentially activate the antioxidant defense systems in human hepatocytes. In moderate hyperglycemia, insulin upregulated CAT expression without affecting SOD, while FFAs upregulated both enzymes. By contrast, in severe hyperglycemia, insulin upregulated CAT but downregulated SOD, and FFAs downregulated CAT without altering SOD. The divergent regulation of antioxidant enzymes highlights the complexity of redox dysregulation in metabolic diseases with context-dependent vulnerabilities in antioxidant defenses. These findings are consistent with reports showing that hyperglycemia triggers a generalized antioxidant response [47], while insulin preferentially enhances H_2_O_2_ detoxification [48]. Our findings indicate that the combination of glucose and FFA excess exacerbates oxidative damage. Notably, the increase in GSK-3β mRNA across all conditions is consistent with its established role in oxidative stress adaptation [49]. In fact, GSK-3β is transcriptionally upregulated in response to oxidative stress, and it functions as a key regulator of the antioxidant response [50,51].

In the liver, oxidative stress, inflammation, and metabolic homeostasis are also controlled by SIRT1, acting as a cellular sensor for energy availability [52]. We observed that in control conditions (MG), either insulin or FFAs downregulated SIRT1 mRNA, whereas under HG conditions, insulin partially restored SIRT1 expression and FFAs further exacerbated its downregulation. Interestingly, these results are in line with data showing that in both isolated cells and mice with IR, SIRT1 expression was downregulated [53], whereas hepatic SIRT1 knockdown led to mild hypoglycemia, increased insulin sensitivity, and decreased glucose synthesis [54].

We also assessed insulin receptor expression and observed that the effects of insulin and free fatty acids (FFAs) are primarily modulated by glucose concentrations. In fact, severe hyperglycemia downregulated InsR expression, as well as insulin and FFAs under moderate hyperglycemia. Instead, under severe hyperglycemia, both insulin and FFAs upregulated InsR levels. This is consistent with recent findings that chronic hyperinsulinemia directly downregulates insulin receptor availability through a negative feedback loop [55], that free fatty acids decrease hepatocyte insulin receptors via PKC-δ activation [56], and that hyperglycemia itself may trigger insulin receptor downregulation as a protective mechanism against glucotoxicity [57].

Insulin signaling through InsR activates a downstream signaling cascade [58], which we assessed by measuring AKT phosphorylation at Ser473. Under moderate hyperglycemia, insulin increased pAKT despite reducing insulin receptor levels, consistent with pathway-selective IR, where certain AKT-dependent signaling branches remain functional and others are impaired [59]. Likewise, FFAs under moderate hyperglycemia increased pAKT despite reduced insulin receptor expression, consistent with reports suggesting that palmitate may induce “insulin-like” effects [60]. Conversely, severe hyperglycemia alone did not increase pAKT despite insulin receptor downregulation, consistent with chronic hyperglycemia impairing insulin signaling via serine kinase-mediated IRS-1 inhibition [61]. However, under severe hyperglycemia, both insulin and fatty acids robustly increased pAKT, with fatty acids producing the highest activation and restoring insulin receptor expression [18]. These results indicate that hyperinsulinemia and FFAs promote pAKT activation through distinct mechanisms, receptor downregulation and sustained signaling activation, respectively, and that their combination with severe hyperglycemia generates a unique IR phenotype.

We wish to underline several limitations of this study. First, the experiments were performed in HepG_2_ cells, a hepatoma-derived line that, while widely used in metabolic research, does not fully replicate the physiology of primary human hepatocytes, particularly due to altered gluconeogenic enzyme expression and insulin signaling, limiting direct extrapolation to physiological liver function [62]. Second, the 24 h treatment window captures early adaptive and maladaptive responses but does not reflect the chronic, progressive development of insulin resistance in vivo. Within this timeframe, we observed a transition from initial insulin responsiveness, evidenced by increased AKT phosphorylation and glycogen accumulation, to a state of metabolic saturation and unresponsiveness. Whether these rapid adaptations translate to long-term pathophysiological mechanisms remains to be determined. Third, both 25 and 50 mM glucose may potentially induce osmotic stress, and, in fact, in conditions like diabetes mellitus, elevated glucose levels typically lead to an increase in serum osmolality. Although osmotic control (e.g., mannitol) was not included in the present study, we wish to emphasize that the MTT assay we performed ensured that the increased osmolarity induced by both the moderate and severe glucose excess did not affect the cell viability of HepG_2_ cells. We wish to note that previous studies [16,63] on the effects of high glucose on HepG_2_ cells were also conducted without osmotic controls. In future studies, experiments with primary human hepatocytes, extended exposure protocols, comprehensive signaling pathway analyses at both mRNA and protein levels, and osmotic stress investigations could be used to validate and extend the present findings.

## 5. Conclusions

In conclusion, our results demonstrate that hyperinsulinemia under both moderate and severe glucose excess effectively induces hepatic insulin resistance, while other combined metabolic insults trigger varying degrees of IR phenotypes in hepatic cells. More broadly, these findings suggest that distinct metabolic triggers can converge on context-dependent adaptive states, each characterized by unique transcriptional and metabolic signatures that collectively contribute to hepatic insulin resistance. Notably, in vitro insulin resistance is not a global failure of insulin signaling but rather a context-specific metabolic reprogramming. This is reflected by paradoxical signaling activation alongside impaired glucose utilization and by glucose-dependent modulation of key metabolic regulators, including PCK1, GSK-3β, PPARγ, and SIRT1, as well as the oxidative stress response, establishing a self-perpetuating cycle of dysfunction. The apparent inconsistencies in pAKT, glycogen, and PCK1 across conditions reflect the dynamic and transitional nature of the adaptive state captured in our model, reinforcing that insulin resistance is a progressive metabolic remodeling rather than a static or uniform failure. We anticipate that the cellular platform established here may facilitate future studies aimed at precisely targeting downstream pathways according to the primary metabolic insult.

## Figures and Tables

**Table 1:** Sequences of the primer pairs used for quantitative real-time PCR. Primer pairs were custom designed specifically for human genes based on their coding sequences, which were obtained from the NCBI GenBank database.

Gene	Forward Primer 5′ ---> 3′	Reverse Primer 5′ ---> 3′	Gene Accession Number
GAPDH	ACC CAC TCC TCC ACC TTT GAC GC	CTC TTG TGC TCT TGC TGG GGC TG	NM-002046.3
SOD	GGC CGT GTG CGT GCT GAA GG	CCC CAC ACC TTC ACT GGT CC	NM-000454.5
CAT	GGG GGA TTC CAG ATG GAC ATC G	CCT GGG AAA GTC TCG CCG CAT C	NM-001752.4
SIRT-1	CAG TGG CTG GAA CAG TGA G	GTC CCA AAT CCA GCT CCT C	NM-012238.5
PPARγ	CGA AGA CAT TCC ATT CAC AAG	CTC CAC AGA CAC GAC ATT C	NM-138711.6
GSK-3β	GAA ATG GAT CAT TTG GTG TG	ACG CAA TCG GAC TAT GTT AC	NM-001146156.2
PEPCK	TGA AGG CAT TAT CTT TGG AG	CGA AGT TGT AGC CAA AGA AG	NM-002591.4

## Data Availability

The data sets generated and/or analyzed during the current study are available from the corresponding author upon reasonable request.

## Supplementary Materials

The following supporting information can be downloaded at https://www.mdpi.com/article/10.3390/cimb48070729/s1.

## Abbreviations

The following abbreviations are used in this manuscript:

Akt	Protein Kinase B
ATCC	American Type Culture Collection
BG	Physiological Glucose Concentration
BSA	Bovine Serum Albumin
CAT	Catalase
CVD	Cardiovascular Disease
DCF	2′,7′-Dichlorofluorescein
DCF-DA	2′,7′-Dichlorofluorescin Diacetate
DFI	DCF Fluorescence Intensity
DTT	Dithiothreitol
ECL	Enhanced Chemiluminescence
EDTA	Ethylenediaminetetraacetic acid
EMEM	Eagle’s Minimum Essential Medium
FBS	Fetal Bovine Serum
FCS	Fetal Calf Serum
FFAs	Free Fatty Acids
GAPDH	Glyceraldehyde 3-Phosphate Dehydrogenase
GSK-3	Glycogen Synthase Kinase-3
HG	Severe Hyperglycemia Condition
HG + OP	Severe Hyperglycemia + Oleate/Palmitate Condition
HRP	Horseradish Peroxidase
InsR	Insulin Receptor
IR	Insulin Resistance
LDs	Lipid Droplets
MASLD	Metabolic Dysfunction-Associated Steatotic Liver Disease
MG	Moderate Hyperglycemia Condition
MG + OP	Moderate Hyperglycemia + Oleate/Palmitate Condition
MTT	3-(4,5-Dimethylthiazol-2-yl)-2,5-Diphenyltetrazolium Bromide
NF-κB	Nuclear Factor Kappa-Light-Chain-Enhancer of Activated B Cells
ORO	Oil Red O
PBS	Phosphate-Buffered Saline
PBS-T	PBS with Tween 20
PCK1	Phosphoenolpyruvate Carboxy Kinase 1
PI3K	Phosphoinositide 3-Kinase
PMSF	Phenylmethylsulfonyl Fluoride
PPARγ	Peroxisome Proliferator-Activated Receptor Gamma
qPCR	Quantitative Real-Time Polymerase Chain Reaction
ROS	Reactive Oxygen Species
SDS	Sodium Dodecyl Sulfate
SIRT1	Sirtuin 1
SOD	Superoxide Dismutase
T2DM	Type 2 Diabetes Mellitus
TAGs	Triacylglycerols
