# Supplementary material for: Metabolic and Signaling Dysregulation in a Cellular Model of Hepatic Insulin Resistance
Source: Curr Issues Mol Biol. 2026 Jul 17;48(7):729. doi: 10.3390/cimb48070729 (PMC13408292; doi:10.3390/cimb48070729)
Supplement: Supplementary file 1 [file cimb-48-00729-s001.zip › cimb-4400365-supplementary.pdf]

Supplementary Materials

Metabolic and Signaling Dysregulation in a Cellular Model of Hepatic Insulin Resistance

Hawraa Zbeeb [1,2], Chourouk Joumaa [1], Giulia De Negri Atanasio [1], Alberto Diaspro [2,3] and Laura Vergani [1,*]

[1] Department of Earth, Environment and Life Sciences (DISTAV), University of Genova, Corso Europa 26, 16132 Genova, Italy; hawraa.zbeeb@iit.it (H.Z.); joumaa.chourouk@edu.unige.it (C.J.); giulia.denegri@edu.unige.it (G.D.N.A.)

[2] Nanoscopy and NIC@IIT, Istituto Italiano di Tecnologia (IIT), via Enrico melen 83, 16153 Genova, Italy; alberto.diaspro@iit.it

[3] Department of Physics (DIFI), University of Genova,via Dodecaneso 33, 16146 Genova, Italy

* Correspondence: laura.vergani@unige.it; Tel.: +39-010-3538403; Fax: +39-010-3538067

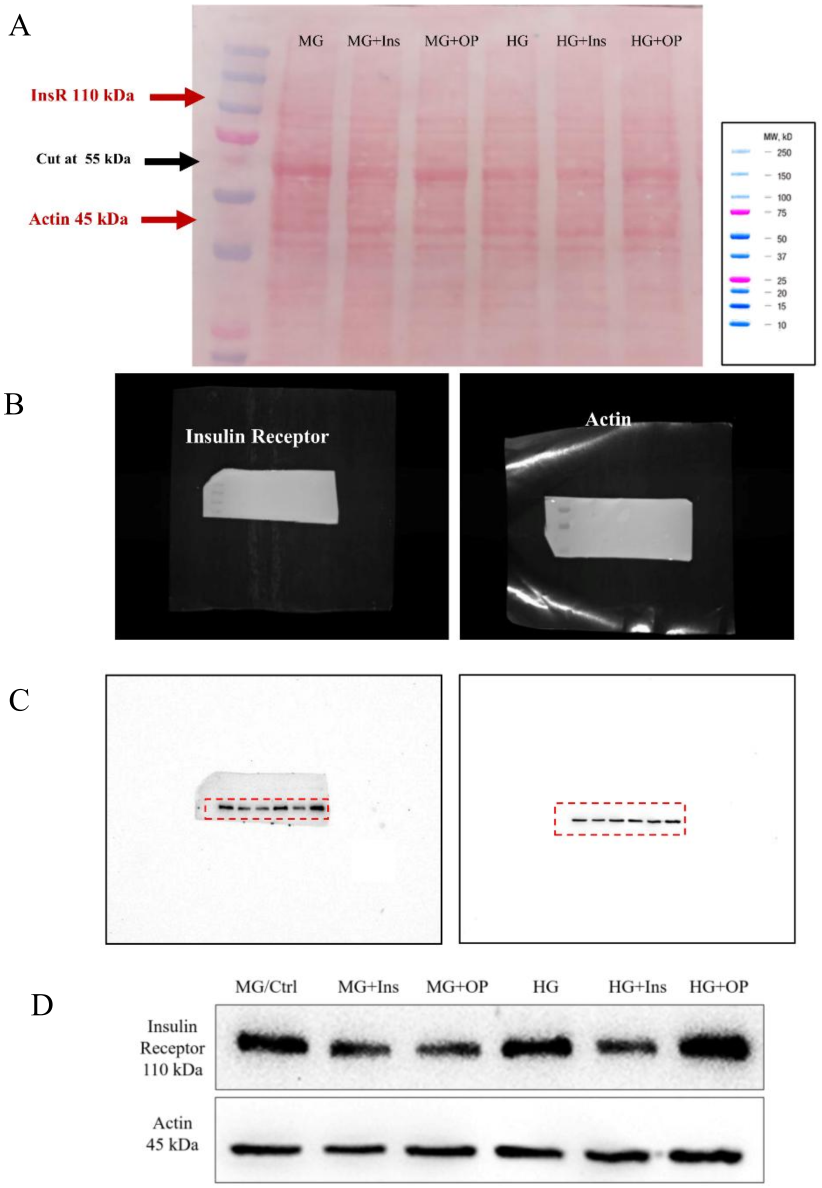

Figure S1. Full, uncropped immunoblots corresponding to Figure 6B. The Western blot procedure and corresponding images are shown for three independent replicates. (A) Full membranes stained with Ponceau S after transfer. (B) Membrane sections for insulin receptor (InsR) and actin under white light, captured immediately before chemiluminescent imaging. (C) Corresponding chemiluminescent signals acquired by the ChemiDoc system. Red dashed boxes indicate the regions cropped for presentation in the main figure (Fig. 6B). (D) The resulting representative bands. To enable simultaneous probing, membranes were cut horizontally at ~55 kDa following transfer. The molecular weight ladder (in kDa) is shown on the left (panels A and B). The predicted molecular sizes are approximately 110 kDa for InsR and 45 kDa for actin. For clarity in the main figure, contrast and brightness were uniformly adjusted across the entire chemiluminescent images (panel C). Two additional independent replicates are also provided.
